# Hubs, influencers, and communities of executive functions: a task-based fMRI graph analysis

**DOI:** 10.3389/fnhum.2025.1525497

**Published:** 2025-08-25

**Authors:** Alexandra T. Davis

**Affiliations:** Baptist Medical Center, Department of Behavioral Health, Jacksonville, FL, United States

**Keywords:** graph theory, connectome, executive functioning, brain network, graph analysis

## Abstract

**Introduction:**

This study investigates four subdomains of executive functioning—initiation, cognitive inhibition, mental shifting, and working memory—using task-based functional magnetic resonance imaging (fMRI) data and graph analysis.

**Methods:**

We used healthy adults’ functional magnetic resonance imaging (fMRI) data to construct brain connectomes and network graphs for each task and analyzed global and node-level graph metrics.

**Results:**

The bilateral precuneus and right medial prefrontal cortex emerged as pivotal hubs and influencers, emphasizing their crucial regulatory role in all four subdomains of executive function. Furthermore, distinct hubs and influencers were identified in cognitive inhibition and mental shifting tasks, elucidating unique network dynamics. Our results suggest a decentralized brain organization with critical hub regions pertinent to conditions such as stroke and traumatic brain injury.

**Discussion:**

The precuneus and medial prefrontal cortex stand out as consistent, domain-general nodes in our findings, which show both unique and shared neural hubs across executive function subdomains. The presence of distinct hubs in cognitive inhibition and mental shifting tasks suggests flexible, task-specific network configurations. A decentralized yet structured brain network may also promote cognitive resilience.

## Introduction

Executive functioning refers to several mental processes vital to effective cognitive control, encompassing tasks such as planning and organizing, initiation, time management, task shifting, and emotion regulation (Friedman and Robbins, 2022; Nemeth and Chustz, 2020). The current study utilizes archival fMRI data on initiation to investigate four subdomains: initiation, cognitive inhibition, mental shifting, and working memory.

Initiation, associated with the dorsolateral prefrontal cortex (DLPFC) and anterior cingulate cortex (ACC), involves starting and executing actions or cognitive processes (Jobson et al., 2021; Friedman and Robbins, 2022; Menon and D’Esposito, 2022). Cognitive inhibition, or inhibitory control, involves the inferior frontal gyrus, insula, superior parietal lobule (SPL), and middle cingulate (Long et al., 2022), while response inhibition involves the anterior cingulate cortex (ACC) and the pre-supplementary motor area (pre-SMA) (Morein-Zamir et al., 2013). Mental shifting, tied to the DLPFC and parietal cortex, facilitates flexible cognitive switching between or among tasks (Friedman and Robbins, 2022; Menon and D’Esposito, 2022). Working memory involves the temporary storage and manipulation of information, engaging a distributed network of brain regions, including the dorsolateral prefrontal cortex (DLPFC), the posterior parietal cortex (PPC), and the ventrolateral prefrontal cortex (VLPFC) (Chai et al., 2018; Engstrom et al., 2013).

Cognitive paradigms, such as the go/no-go, local task-switching, and n-back tasks, systematically investigate the four subdomains of executive function. The go/no-go paradigm assesses initiation and inhibition, elucidating the crucial connection between executive functioning and inhibitory control, which are indispensable for goal-directed behavior (Diamond, 2013; Verbruggen and Logan, 2008). Similarly, the local task-switching paradigm evaluates cognitive flexibility by observing how individuals manage attentional resources when transitioning between tasks with shared characteristics (Huff et al., 2015). Finally, the n-back paradigm, mainly the 2-back task, challenges participants with recalling and matching stimuli across a sequence (Jaeggi et al., 2010; Niendam et al., 2012). By employing cognitive paradigms, the present study delves into graph analysis metrics that assess a network graph’s structure, connectivity, and relationships, explicitly focusing on initiation, cognitive inhibition, shifting, and working memory.

Investigations into executive functioning often concentrate on specific brain regions (Friedman and Robbins, 2022; Menon and D’Esposito, 2022) or adopt a topographical network perspective that may use inconsistent labels for overall executive control abilities (Witt et al., 2021). In contrast, our study utilizes graph-based network analysis techniques to explore well-defined subdomains of executive function. This approach offers a robust framework for understanding the brain’s interconnected networks, effectively addressing the limitations of traditional region-based methods in studying executive functions.

While earlier research frequently focused on isolated regions, such as the prefrontal cortex, graph theory allows for examining brain-wide interactions, revealing emergent properties like global efficiency (communication efficiency across network nodes) and modularity (network structure strength) (Bullmore and Sporns, 2012; Rubinov and Sporns, 2010). The graph-based network perspective emphasizes the significance of relationships among brain regions. This approach provides insights into functional connectivity and dynamics by representing interactions as graphs. A significant advantage of this method is its capacity to identify critical nodes (hubs and influencers), similar to a social network. These regions are essential for network communication and integration. In the context of executive functions, this reveals vital components often overlooked in region-specific studies (Medaglia et al., 2015; Baum et al., 2017).

Moreover, graph theory facilitates task-specific comparisons, illustrating how different executive functions recruit distinct or overlapping network features, such as community structure or task flexibility. It quantifies the balance between functional integration (efficient global communication) and segregation (specialized local processing), which are crucial metrics for understanding brain organization during executive tasks (Baum et al., 2017; Ramos-Nuñez et al., 2017). Graph theory also sheds light on disruptions observed in clinical populations by linking network-level metrics to individual differences in cognitive performance.

This holistic approach captures both local and global properties of brain interactions, filling the gaps left by traditional region-specific analyses. It advances our understanding of the neural basis of executive functions by focusing on system-wide organization rather than isolated activity. Despite the growing interest in this area, few studies have systematically compared these subdomains using network-based graph theory approaches. Our research aims to bridge this gap by integrating traditional regional methods with graph analyses, providing a more comprehensive understanding of executive functions.

Graph-based network analysis serves as a valuable framework for unraveling the complexities inherent in systems represented as graphs. This methodology enhances our comprehension of intricate brain network connectivities and patterns (Bullmore and Sporns, 2009). Certain cortical areas emerge as highly connected or centralized regions, critical focal points (Farahani et al., 2019). The application of graph-based theory in cognitive neuroscience, particularly in human connectome studies, has evolved significantly, correlating brain network properties with human intelligence, memory, attention, and emotional processing (Farahani et al., 2019). For instance, research has demonstrated a correlation between working memory performance and local/global measures in brain networks (Stanley et al., 2015). Additionally, disruptions in functional network topology have been implicated in various cognitive and psychiatric disorders (Reijneveld et al., 2007).

This study aims to investigate core graph metrics to understand the network properties of cortical areas crucial for executive functions, specifically initiation, inhibition, shifting, and working memory in healthy adults. Utilizing graph-based network analysis, we seek to address several critical research questions:

1. Are there significant differences in brain network graphs between these executive functions?
2. What specific network features are associated with each task?
3. Which brain regions are essential for these functions?

Moreover, we propose the following hypotheses:

1. Brain network graphs will display significant differences in their topological properties—such as clustering coefficient, modularity, and global efficiency—across tasks related to different executive functions. This variation will reflect the distinct neural processing demands of each task.
2. Each executive function task will yield unique network features. For instance, tasks emphasizing working memory are expected to exhibit higher modularity, while those involving cognitive inhibition will show increased connectivity in control-related regions. Additionally, we anticipate tasks focused on shifting demonstrate greater flexibility in inter-community connections.
3. Specific brain regions will serve as critical hubs or influencers across these tasks. We expect the dorsolateral prefrontal cortex to play a central role in working memory, the anterior cingulate cortex vital for cognitive inhibition, and the parietal regions to be key in task-shifting. These essential regions likely exhibit high centrality and betweenness values, underscoring their importance in network communication.

This study aims to enhance our understanding of the neural underpinnings of executive functioning and its relationship with brain network organization by addressing these questions.

## Methods

### Data acquisition

This study employed a publicly available dataset derived from functional magnetic resonance imaging (fMRI) scans of healthy adults (Rieck et al., 2021). One hundred forty-four participants (ages 20 to 86) underwent scanning using a Siemens 3 T MRI scanner while engaging in cognitive paradigms to assess functional activity. These paradigms included a go/no-go task for examining inhibition and initiation, a local task-switching paradigm for shifting, and an n-back task with three load levels (0-back, 1-back, and 2-back) for working memory (Rieck et al., 2021).

Functional connectivity estimates (quantified with time-series correlations) between various brain regions were computed using three distinct brain atlases (Rieck et al., 2021): the Schaefer 100 parcel 17 network atlas (Schaefer et al., 2018; Thomas Yeo et al., 2011), the Power 229 node 10 network atlas (Power et al., 2011), and the Schaefer 200 parcel 17 network atlas (Schaefer et al., 2018; Thomas Yeo et al., 2011; Rieck et al., 2021). The present study utilizes the correlation data obtained from the Schaefer 200 parcel 17 network atlas.

### Processing

To identify relevant brain regions (ROIs) with robust functional connectivity, ROIs exhibiting a high correlation coefficient exceeding 0.75 in the adjacency matrices of individual participants were selected. ROIs associated with the motor and visual networks were excluded to maintain the study’s focus on executive functioning. Additionally, ROIs with limited occurrences—those with connections observed in less than 10 participants—were also excluded to ensure the inclusion of reliably connected brain regions. The resulting ROIs and the corresponding aggregated frequency of functional connectivity incidents constitute an adjacency matrix for each task (inhibition, initiation, shifting, and 2-back).

### Brain network construction

**Figure 1:**
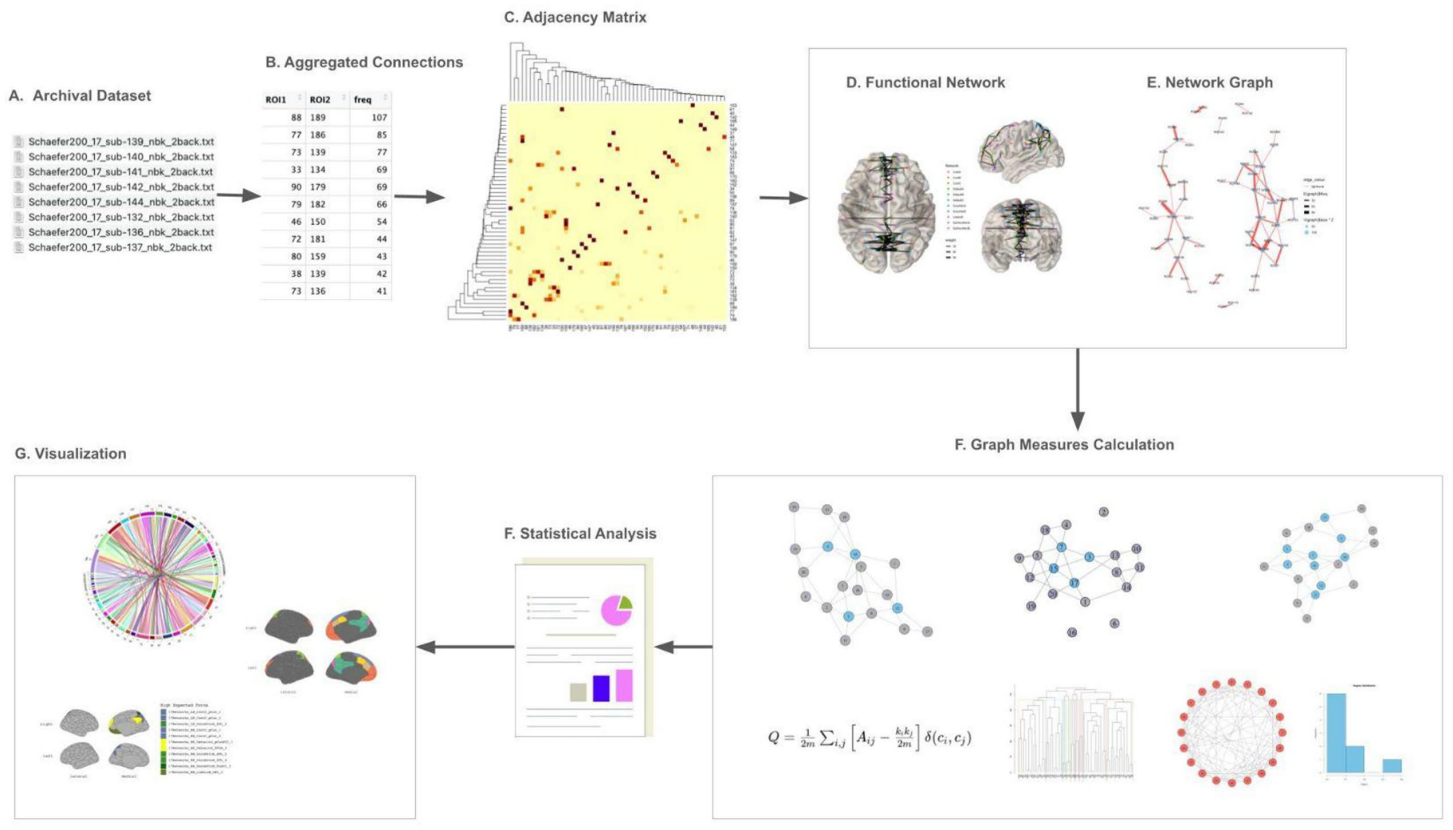
Pipeline for data analysis. **(A)** Obtain raw archival data. **(B)** Functional connectivity matrices are constructed, and connections with less frequency were excluded. **(C)** Adjacency matrices were derived based on step **(B)**. **(D)** Graphs were generated and plotted on connectome. **(E)** Graph theoretical analysis. Metrics such as degree centrality, clustering coefficient, and modularity are computed to characterize the network’s local and global properties. **(F)** Statistical analyses are applied to identify significant patterns or differences across tasks. **(G)** Results were plotted on Schaefer for visualizations of the findings.

**Figure 2:**
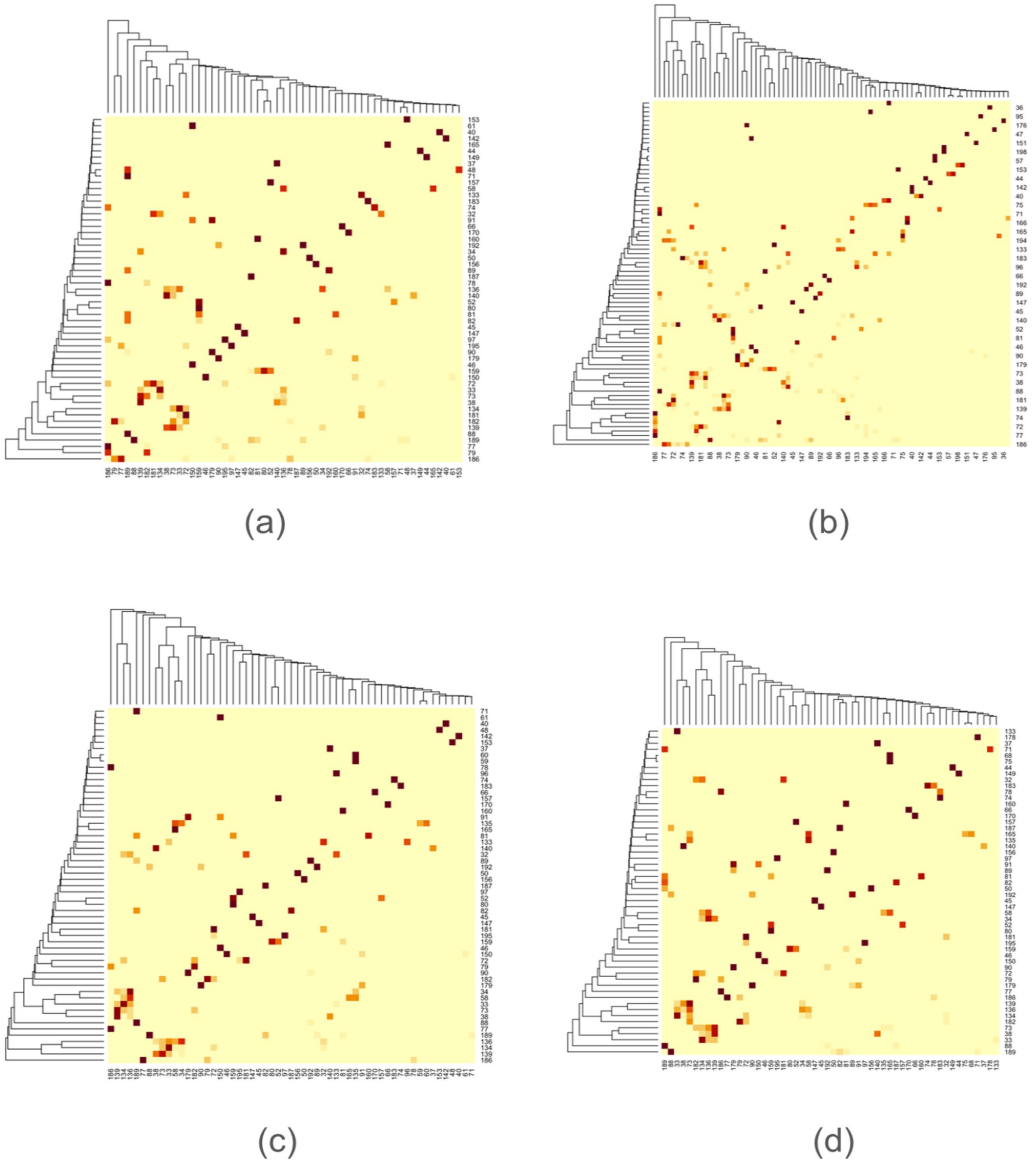
Adjacency matrix for each task. **(a)** Initiation, **(b)** Inhibition, **(c)** Shifting, **(d)** 2-back.

**Figure 3:**
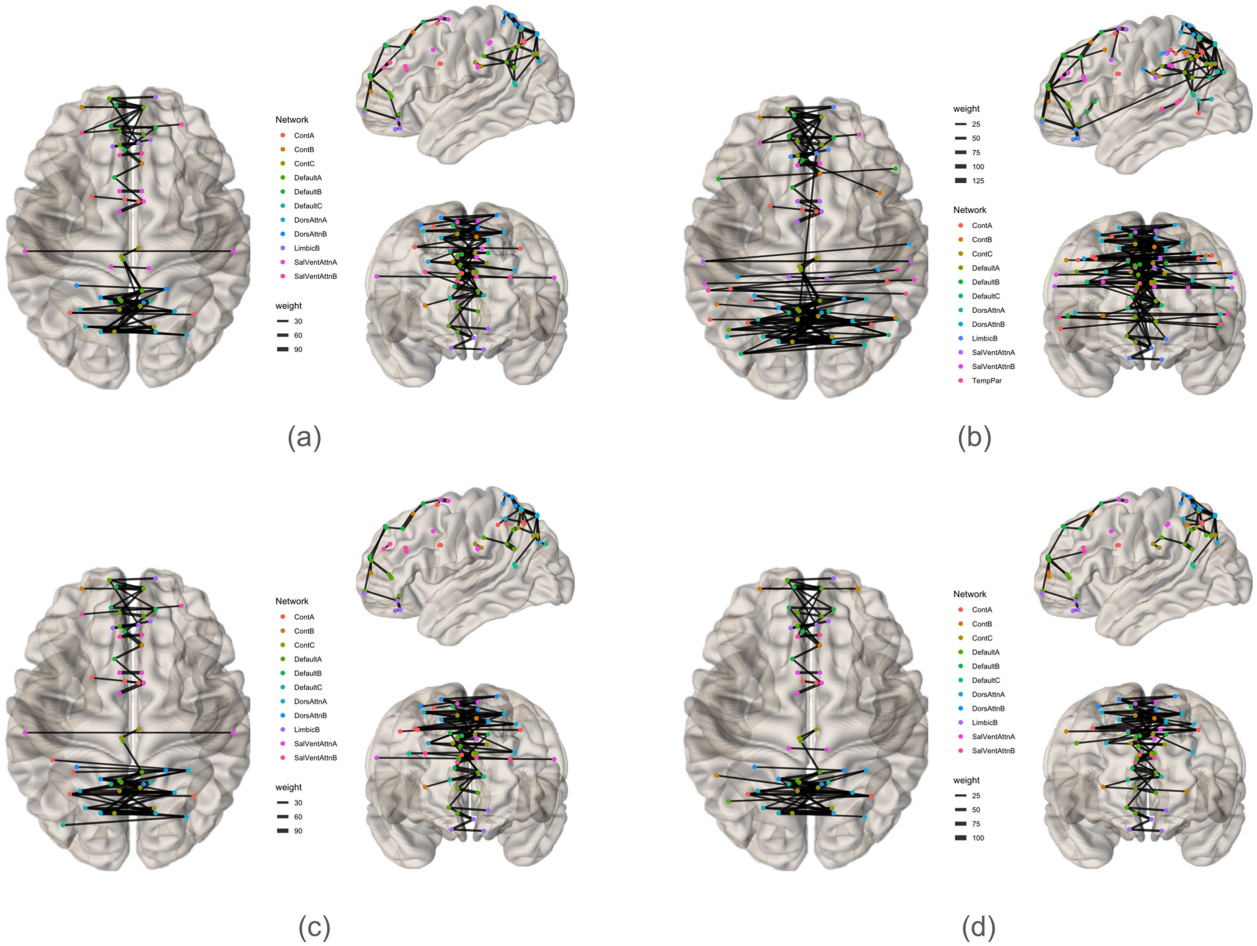
Graphs plotted on connectome for each task. **(a)** Initiation, **(b)** Inhibition, **(c)** Shifting, **(d)** 2-back.

**Figure 4:**
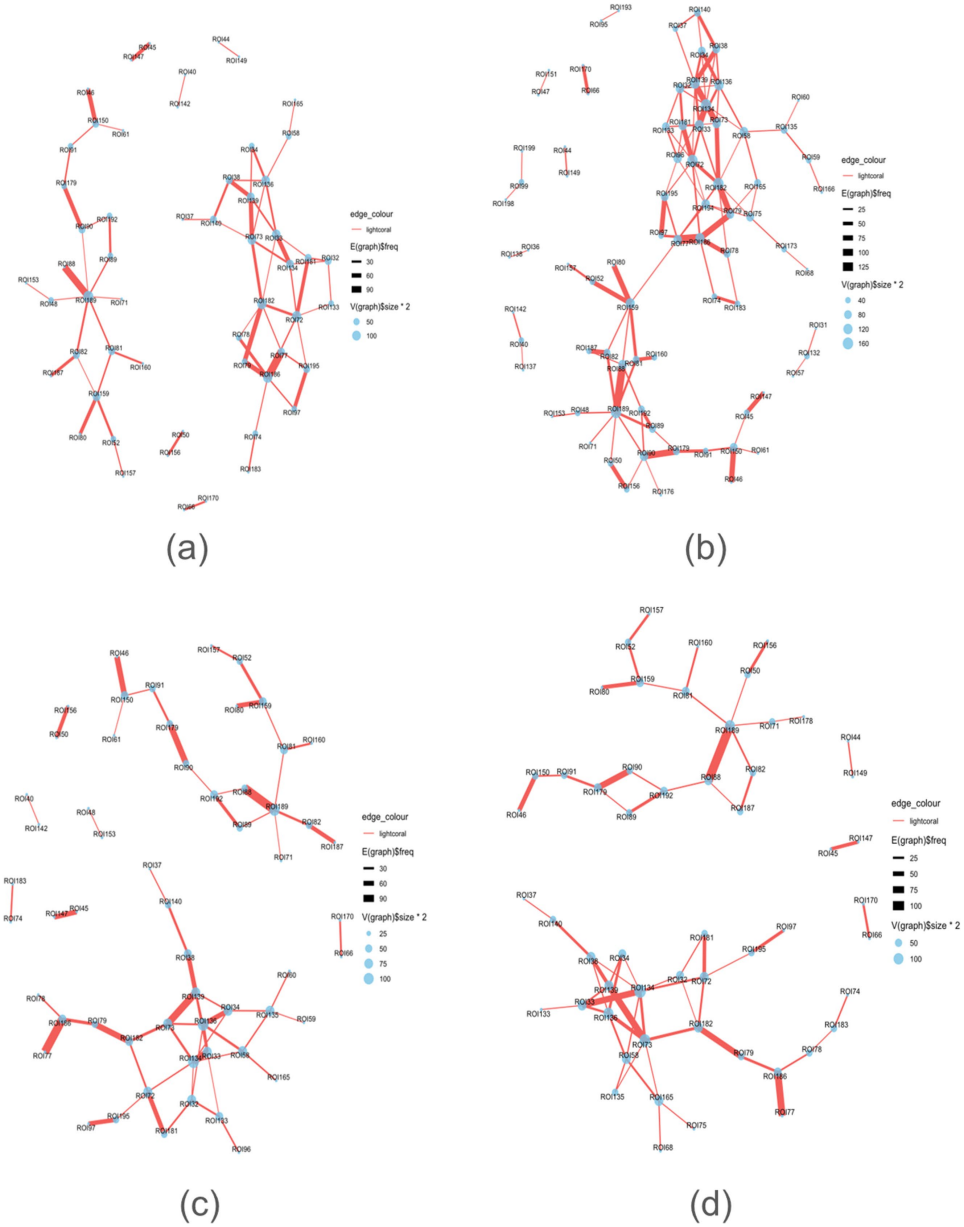
Graphs for each task. **(a)** Initiation, **(b)** Inhibition, **(c)** Shifting, **(d)** 2-back.

The analysis pipeline is depicted in Figure 1. A brain graph network comprises nodes (brain regions) and edges (functional connectivities) (Fornito et al., 2016). Nodes can be assigned binary or weighted values representing activity intensity. Considering the interindividual variability in brain connectomes (Sun et al., 2022), we aggregate weighted values across participants to obtain collective brain activity. This connectivity was utilized to construct an adjacency matrix (Figure 2), which signifies connections between nodes in a graph (Alper et al., 2013). The functional network was mapped using an adjacency matrix for each task and visualized on a connectome utilizing the Schaefer200_n17 atlas (Figures 2, 3). Furthermore, network graphs were generated for each task (Figure 4).

### Graph measures

Due to a lack of directionality in fMRI data, the current study employs weighted undirected matrices and investigates the topological characteristics of functional brain networks for each task. To analyze topographical features, conventional graph measures such as node centrality measures (degree, strength, betweenness, and closeness), clustering coefficient, modularity, characteristic path length, and small-worldedness, among others, were employed (Tables 1–5) (Sporns and Betzel, 2016; van den Heuvel et al., 2008).

**Table 1:** Node degree.

Initiation		Inhibition		Shifting		2-back	
Node	Degree	Node	Degree	Node	Degree	Node	Degree
189	7	72	8	134	6	134	7
72	6	189	8	136	6	73	6
182	6	134	8	189	5	189	5
186	6	182	8	73	4	136	5
73	5	139	7	33	4	33	4
33	5	33	6	34	4	72	4
136	5	186	6	72	4	38	4
139	4	159	6	58	4	58	4
134	4	136	6	32	4	139	4
159	4	77	5	139	4	182	4
77	3	88	5	135	4	165	4
195	3	90	5	38	3	88	3
82	3	73	5	81	3	34	3
81	3	32	5	186	3	81	3
140	3	58	5	150	3	32	3
38	3	96	5	182	3	186	3
150	3	181	5	159	3	179	3
90	3	194	5	192	3	159	3
32	3	79	4	133	3	192	3
181	3	38	4	88	2	90	2
78	2	34	4	90	2	79	2
79	2	75	4	79	2	50	2
179	2	150	4	82	2	89	2
97	2	195	4	89	2	52	2
133	2	133	4	52	2	82	2
74	2	82	3	91	2	91	2
192	2	78	3	179	2	78	2
34	2	81	3	181	2	71	2
52	2	89	3	195	2	150	2
89	2	179	3	140	2	181	2
91	2	140	3	77	1	195	2
58	2	192	3	46	1	140	2
48	2	165	3	45	1	187	2
147	1	135	3	80	1	183	2
44	1	97	2	97	1	135	2
61	1	45	2	50	1	77	1
183	1	52	2	66	1	46	1
170	1	50	2	74	1	80	1
40	1	74	2	96	1	45	1
160	1	91	2	78	1	97	1
50	1	40	2	60	1	66	1
66	1	37	2	37	1	74	1
156	1	48	2	40	1	44	1
187	1	59	2	48	1	68	1
142	1	99	2	59	1	37	1
80	1	187	2	61	1	75	1
45	1	156	2	71	1	147	1
46	1	160	2	147	1	156	1
88	1	183	2	187	1	157	1
37	1	173	2	156	1	170	1
71	1	132	2	160	1	160	1
157	1	46	1	157	1	149	1
149	1	80	1	170	1	133	1
165	1	66	1	183	1	178	1
153	1	44	1	165	1		
		71	1	142	1		
		31	1	153	1		
		47	1				
		61	1				
		36	1				
		57	1				
		60	1				
		68	1				
		95	1				
		147	1				
		170	1				
		157	1				
		142	1				
		149	1				
		153	1				
		166	1				
		199	1				
		151	1				
		138	1				
		137	1				
		176	1				
		193	1				
		198	1				

**Table 2:** Node strength.

Initiation		Inhibition		Shifting		2-back	
Node	Strength	Node	Strength	Node	Strength	Node	Strength
186	286	186	365	139	208	73	205
189	205	189	300	136	187	134	174
182	186	182	291	73	185	189	167
139	168	139	275	189	182	139	158
73	162	159	244	186	176	136	134
77	155	73	233	134	176	186	133
72	152	134	232	33	168	88	132
33	142	33	231	182	136	182	132
79	139	72	227	88	125	33	125
134	124	79	210	77	117	179	108
159	122	77	203	72	117	72	102
38	110	88	186	79	115	38	97
136	106	181	162	34	114	79	95
88	102	136	161	179	114	77	85
150	93	38	159	159	113	90	84
195	89	90	156	38	111	159	83
90	86	179	151	150	103	58	78
179	86	150	134	90	97	150	72
181	85	81	131	181	85	192	62
82	82	195	125	82	84	165	62
97	75	82	123	58	83	34	61
81	74	34	113	46	79	52	58
46	68	97	112	32	74	181	58
140	59	89	99	195	68	46	54
78	54	52	98	52	65	81	52
147	54	78	94	192	65	82	49
45	54	32	93	45	64	50	46
89	54	192	93	147	64	195	46
80	53	46	87	135	64	89	45
52	53	80	83	80	59	91	45
34	51	140	83	89	56	80	43
192	47	45	80	97	55	140	42
32	46	50	80	133	55	45	39
187	42	187	80	81	54	147	39
156	38	58	76	187	54	187	39
50	38	156	76	50	53	32	36
91	38	133	70	156	53	97	35
74	37	91	68	140	48	156	34
160	28	96	68	91	43	78	30
170	28	147	68	160	26	157	30
66	28	74	63	66	25	183	29
58	25	160	62	157	25	66	28
133	25	183	59	170	25	170	28
183	22	194	59	74	22	135	27
48	21	66	53	183	22	160	24
157	17	170	53	96	21	71	21
37	14	75	47	78	20	74	18
44	13	165	42	165	20	44	17
149	13	135	42	60	17	149	17
142	12	37	36	37	15	68	15
40	12	48	35	40	11	37	12
165	12	59	33	48	11	75	12
153	11	40	31	59	11	133	11
61	10	157	31	142	11	178	10
71	10	173	25	153	11		
		132	24	61	10		
		99	22	71	10		
		142	21				
		44	20				
		149	20				
		153	16				
		71	15				
		31	14				
		166	14				
		199	12				
		47	11				
		61	11				
		151	11				
		36	10				
		57	10				
		60	10				
		68	10				
		95	10				
		138	10				
		137	10				
		176	10				
		193	10				
		198	10				

**Table 3:** Node betweenness.

Initiation		Inhibition		Shifting		2-back	
Node	Betweenness	Node	Betweenness	Node	Betweenness	Node	Betweenness
189	138	159	895.3666667	189	101.5	189	132
182	84.151826	77	848.1891273	134	89.468074	182	132
90	81.5	189	572.5642857	182	88.648268	73	116.2
73	72.513161	182	550.4220114	136	75.97132	134	107.616667
72	68.605463	88	363.0404762	72	73.452381	79	105
179	64	179	312.9166667	192	72.5	88	96.5
186	61.710859	58	265.3270125	73	69.377489	186	91
136	59.64667	91	265	81	69	192	84.5
159	55	90	263.1595238	79	66	81	79
33	54.013823	72	227.7273555	90	65	72	70.9
91	51	150	221	179	56	159	53
81	45	82	189.8714286	58	50.816991	179	51.5
82	45	81	189.8714286	135	47.916667	38	50.733333
150	37	186	180.5773489	186	47	165	50.516667
134	33.551963	89	168.8166667	159	47	78	48
140	24.714596	135	167	38	46.708333	91	36
74	22	194	153.6120443	91	45	90	30
58	22	75	126.5622013	34	40.60303	89	30
48	19	134	125.977697	88	35	136	28.483333
52	19	139	114.5426742	89	35	33	27.733333
139	17.64667	192	111.102381	32	34.75184	195	25
77	14.227234	195	96.4863525	150	33	140	25
181	13.160497	33	76.6583392	139	30.957251	183	25
195	8.555808	73	63.8258992	33	28.289935	32	22.9
78	7.643687	45	57	133	24.791667	58	22.8
79	7.643687	52	57	195	24	50	19
38	6.861111	48	57	140	24	52	19
89	6.5	59	57	82	17	71	19
32	5.341834	173	57	52	17	150	19
192	3	96	54.0447632	181	7.246753	139	18.866667
133	2.861111	136	49.6574909	77	0	82	5.5
97	1.4	78	48.6184711	46	0	187	4
34	0.75	79	33.2078878	45	0	34	2.733333
80	0	38	27.9546775	80	0	135	1.516667
187	0	34	27.9546775	97	0	181	1
44	0	50	20.2404762	50	0	77	0
88	0	165	18.7331633	66	0	46	0
46	0	181	16.4903546	74	0	80	0
71	0	74	13.4422031	96	0	45	0
45	0	133	12.2607623	78	0	97	0
37	0	32	10.9993036	60	0	66	0
50	0	37	6.8630962	37	0	74	0
147	0	156	3.5833333	40	0	44	0
157	0	140	3.1626206	48	0	68	0
66	0	183	2.0774802	59	0	37	0
40	0	97	1.6249851	61	0	75	0
61	0	40	1	71	0	147	0
156	0	99	1	147	0	156	0
170	0	132	1	187	0	157	0
160	0	187	0.7333333	156	0	170	0
183	0	160	0.7333333	160	0	160	0
149	0	46	0	157	0	149	0
142	0	80	0	170	0	133	0
165	0	66	0	183	0	178	0
153	0	44	0	165	0		
		71	0	142	0		
		31	0	153	0		
		47	0				
		61	0				
		36	0				
		57	0				
		60	0				
		68	0				
		95	0				
		147	0				
		170	0				
		157	0				
		142	0				
		149	0				
		153	0				
		166	0				
		199	0				
		151	0				
		138	0				
		137	0				
		176	0				
		193	0				
		198	0				

**Table 4:** Node closeness.

Initiation		Inhibition		Shifting		2-back	
Node	Closeness	Node	Closeness	Node	Closeness	Node	Closeness
156	1	66	1	45	1	45	1
147	1	44	1	50	1	66	1
40	1	47	1	66	1	44	1
45	1	36	1	74	1	147	1
66	1	95	1	40	1	170	1
170	1	170	1	48	1	149	1
149	1	149	1	147	1	189	0.0181818
50	1	151	1	156	1	88	0.0172414
44	1	138	1	170	1	81	0.015625
142	1	193	1	183	1	192	0.0153846
189	0.0217391	40	0.5	142	1	73	0.0151515
182	0.0196078	99	0.5	153	1	134	0.0149254
90	0.0188679	132	0.5	189	0.0181818	182	0.0144928
73	0.0188679	31	0.3333333	88	0.0172414	72	0.0138889
72	0.0181818	57	0.3333333	89	0.0172414	50	0.0138889
82	0.0181818	142	0.3333333	192	0.0163934	82	0.0138889
81	0.0181818	199	0.3333333	81	0.016129	71	0.0138889
33	0.0181818	137	0.3333333	134	0.015873	187	0.0133333
134	0.0181818	198	0.3333333	136	0.015873	90	0.0131579
181	0.0163934	77	0.0049751	73	0.0149254	89	0.0131579
77	0.015873	182	0.0048077	90	0.0147059	32	0.0131579
89	0.015873	159	0.0048077	82	0.0142857	159	0.012987
48	0.015873	186	0.0044248	34	0.0140845	136	0.012987
136	0.015873	194	0.0044248	72	0.0140845	139	0.0126582
159	0.015625	88	0.0044053	58	0.0140845	58	0.0121951
179	0.015625	82	0.004329	139	0.0140845	160	0.0120482
186	0.0153846	81	0.004329	71	0.0138889	79	0.0119048
79	0.0153846	195	0.0042735	33	0.0136986	38	0.0119048
88	0.0153846	72	0.0042553	32	0.0136986	165	0.0117647
78	0.0153846	58	0.0041494	159	0.0136986	33	0.0116279
71	0.0153846	33	0.0041152	182	0.0133333	179	0.0114943
139	0.0149254	73	0.0040816	179	0.012987	34	0.0113636
192	0.0142857	96	0.0040161	160	0.0126582	135	0.011236
140	0.0140845	189	0.004	38	0.0123457	156	0.010989
32	0.0136986	79	0.0039526	181	0.0123457	178	0.010989
195	0.0136986	75	0.0039216	187	0.0114943	52	0.0106383
187	0.0135135	78	0.0038911	135	0.0114943	181	0.0105263
160	0.0135135	192	0.0038168	52	0.0113636	195	0.0105263
133	0.0133333	52	0.0038023	91	0.0113636	80	0.0104167
91	0.012987	134	0.0037879	80	0.0111111	186	0.009901
38	0.0126582	80	0.0037736	133	0.010989	91	0.0098039
52	0.0123457	139	0.0036232	79	0.0107527	140	0.0093458
153	0.0121951	136	0.0035971	195	0.0107527	68	0.0090909
58	0.0120482	97	0.0035842	165	0.0105263	75	0.0090909
80	0.0120482	74	0.0035587	150	0.009901	133	0.009009
34	0.0120482	181	0.0035461	140	0.0097087	157	0.0088496
97	0.0117647	187	0.0035461	157	0.0095238	150	0.0084034
74	0.0117647	160	0.0035461	60	0.009009	97	0.0083333
150	0.0108696	165	0.0035461	59	0.009009	78	0.0081967
37	0.0107527	133	0.0035211	186	0.0088496	77	0.0079365
157	0.01	90	0.0034843	96	0.0086957	37	0.0075758
165	0.0095238	89	0.0034364	97	0.008547	46	0.0072464
183	0.0093458	135	0.0034247	46	0.0084746	183	0.0068966
46	0.009009	50	0.0032787	61	0.0084746	74	0.0058824
61	0.009009	48	0.0032787	37	0.007874		
		71	0.0032573	77	0.0072993		
		173	0.0032258	78	0.0072993		
		32	0.0032154				
		183	0.0032051				
		38	0.0031949				
		34	0.0031949				
		157	0.003125				
		179	0.0030303				
		37	0.0030211				
		156	0.002924				
		176	0.002907				
		59	0.0028818				
		60	0.0028653				
		153	0.0027624				
		140	0.0027322				
		68	0.0027248				
		91	0.0026525				
		166	0.0024752				
		150	0.0023474				
		45	0.002079				
		46	0.0020704				
		61	0.0020704				
		147	0.0018587				

**Table 5:** Graph metrics across four executive tasks and their interpretations.

Metrics	Initiation		Inhibition		Shifting		2-back	
	Value	Interpretation	Value	Interpretation	Value	Interpretation	Value	Interpretation
Modularity	0.66	Network divided into distinct communities	0.62	Network divided into distinct communities	0.68	Network divided into distinct communities	0.69	Network divided into distinct communities
Global Efficiency	0.13	Low efficiency in information transfer	0.16	Moderate efficiency in information transfer	0.12	Low efficiency in information transfer	0.14	Moderate efficiency in information transfer
Path length ratio	0.87	Observed path length is slightly lower than random path length	1.31	Observed path length is about 1.31 times longer than random path length	1.00	Observed path length is very close to random path length	0.96	Observed path length is slightly lower than random path length
Characteristic Path Length	3.35	Nodes are relatively close to each other in terms of network connectivity	5.17	Nodes are relatively distant from each other in terms of network connectivity	3.91	Nodes are relatively close to each other in terms of network connectivity	3.93	Nodes are relatively close to each other in terms of network connectivity
Assortativity	0.07	Slight assortativity, indicating a tendency for nodes with similar degrees to be connected.	0.34	Moderate tendency for nodes to attach to similar nodes	0.37	Moderate tendency for nodes to attach to similar nodes	0.35	Moderate tendency for nodes to attach to similar nodes
Edge Density	0.04	Low, indicating a sparse network with few connections.	0.04	Low, indicating a sparse network with few connections.	0.04	Low, indicating a sparse network with few connections.	0.04	Low, indicating a sparse network with few connections.
Small-worldness (sigma)	0.00	Not exhibiting small-world properties	0.00	Not exhibiting small-world properties	0.00	Not exhibiting small-world properties	0.00	Not exhibiting small-world properties
Transitivity	0.00	Absence of clustering	0.00	Absence of clustering	0.00	Absence of clustering	0.00	Absence of clustering
Clustering Coefficient	0.00	Absence of clustering	0.00	Absence of clustering	0.00	Absence of clustering	0.00	Absence of clustering

While it is frequently challenging to ascertain the most appropriate metrics for investigating brain networks (Bullmore and Sporns, 2009), centrality measures and small-world characteristics (e.g., high clustering coefficient and short characteristic path length) are indispensable for this process (He and Evans, 2010). Although there are no established criteria for “hub status,” most studies consider nodes with high centrality measures as hubs (Farahani et al., 2022; Fornito et al., 2016). In this study, weights denote the aggregate frequency of connections among participants. Local network measures were computed for each node (Brian region), including nodal strength, betweenness centrality, and closeness centrality. Nodes exhibiting the top 20% values for strength and betweenness were designated as hubs.

Furthermore, this study employed a novel centrality measure known as “*expected force*,” which quantifies a node’s potential influence within a network by summing the weights of its connections. This measure identifies critical nodes facilitating information flow, such as key brain regions in executive functions (Bullmore and Bassett, 2011). Nodes exhibiting high expected force are likely to influence other network nodes significantly. Unlike other centrality measures, expected force maintains reliability in network alterations, ensuring accuracy for incomplete or noisy systems (Lawyer, 2015). Therefore, potentially identify brain regions or connections that could be of interest in reorganization post-TBI or seizure disorders. Consequently, the top 20% of nodes with high expected force were identified as influencers.

Global network measures were also computed, including community detection, density, clustering coefficient, modularity, assortativity, characteristic path length, and small-worldedness. Community detection algorithms, such as Louvain and Infomap, identify subnetworks or modules (Hric et al., 2014). Assortativity measures the tendency of nodes in a network to connect with other nodes that have similar or dissimilar properties. In a brain network, it can be used to understand connectivity patterns (Rubinov and Sporns, 2010) and indirectly reflect network resilience (Farahani et al., 2019). Density reflects network connectivity, and modularity assesses community strength. The clustering coefficient indicates node clustering, while characteristic path length gauges information transfer efficiency.

### Statistical analysis

Nonparametric (Kolmogorov–Smirnov) tests were employed to analyze the degree distribution of the graphs. The Kruskal-Wallis test was utilized to compare node strength and hubs across the tasks. In contrast, a pairwise comparison (Wilcoxon rank sum test) was used to elucidate their differences further.

## Results

**Figure 5:**
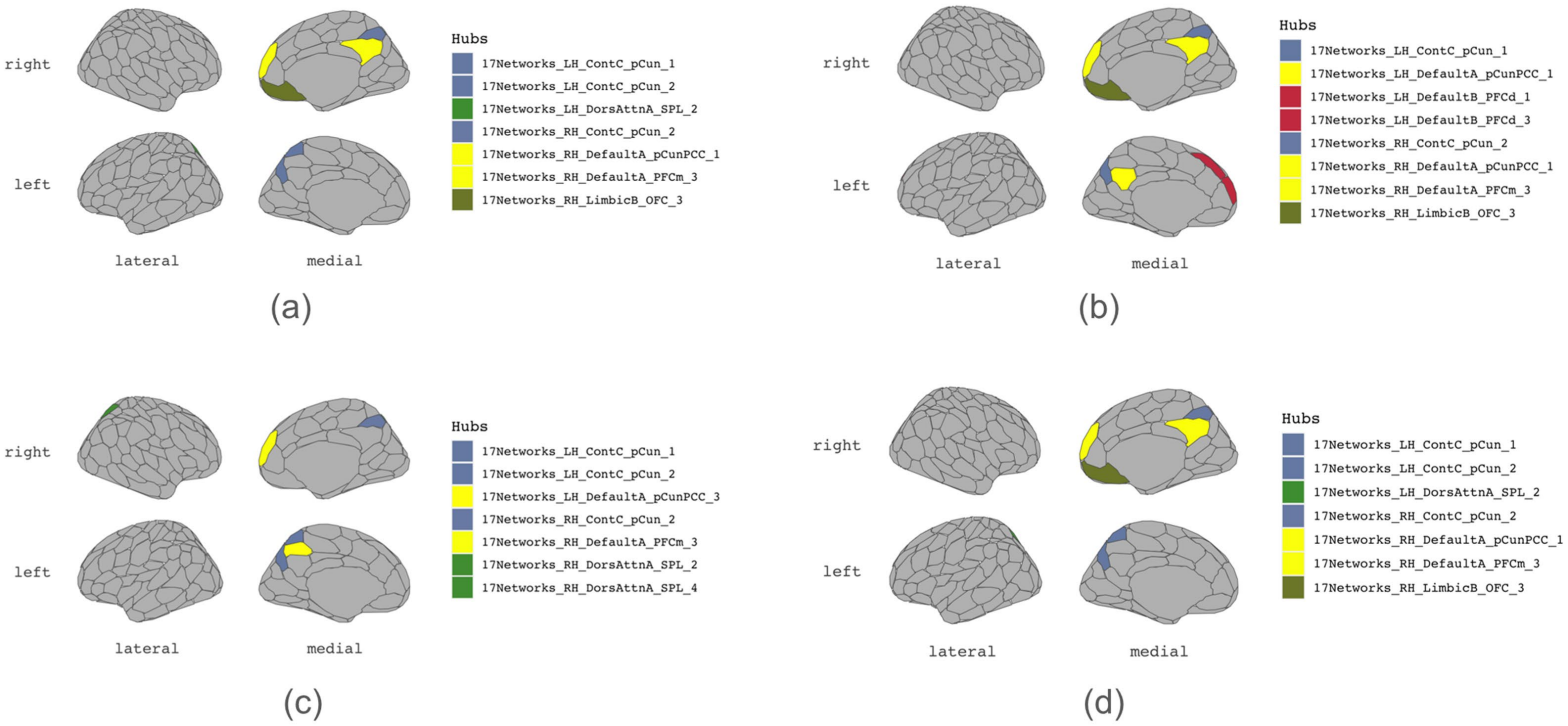
Hubs. **(a)** Initiation, **(b)** Inhibition, **(c)** Shifting, **(d)** 2-back.

**Figure 6:**
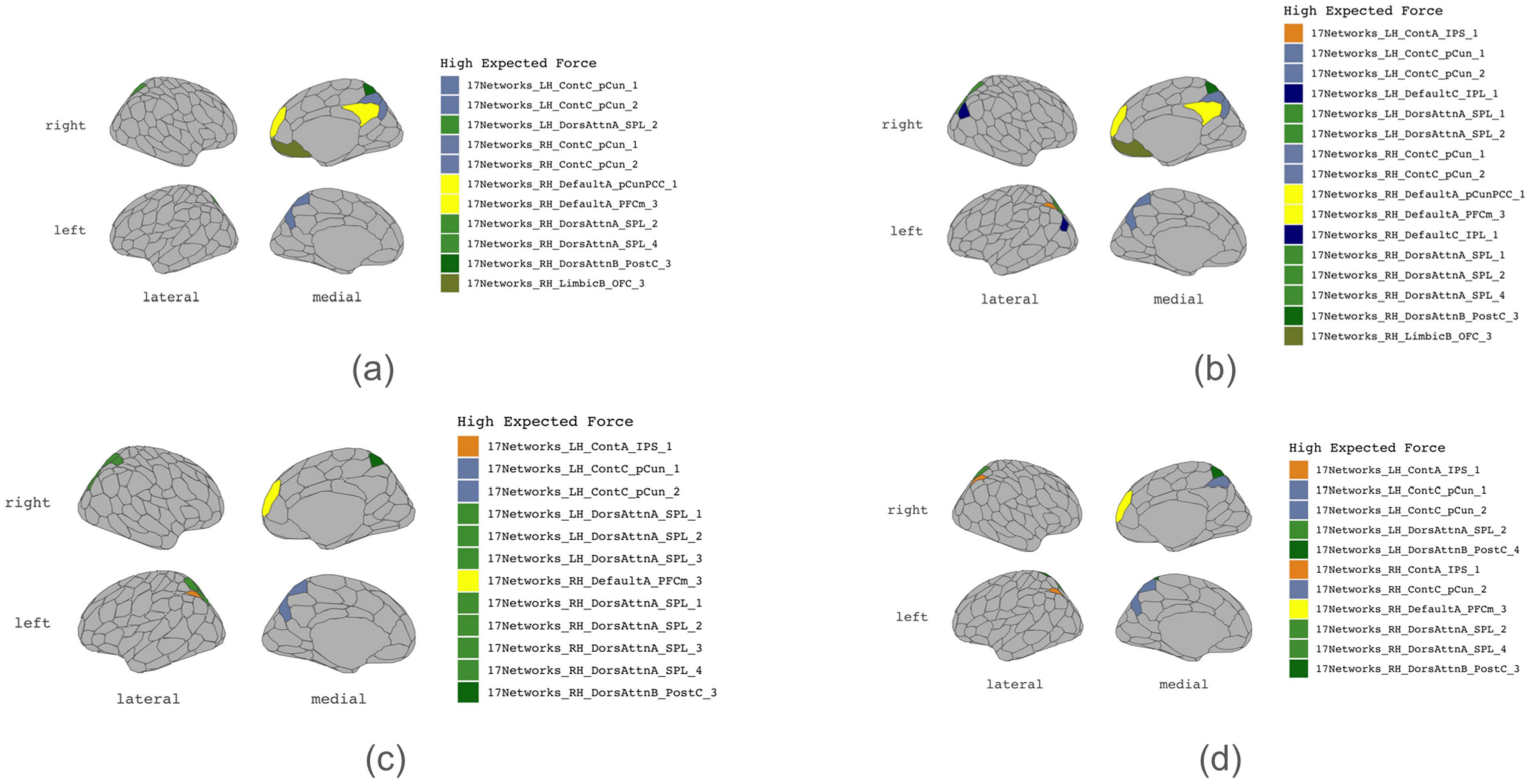
Influencers. **(a)** Initiation, **(b)** Inhibition, **(c)** Shifting, **(d)** 2-back.

**Figure 7:**
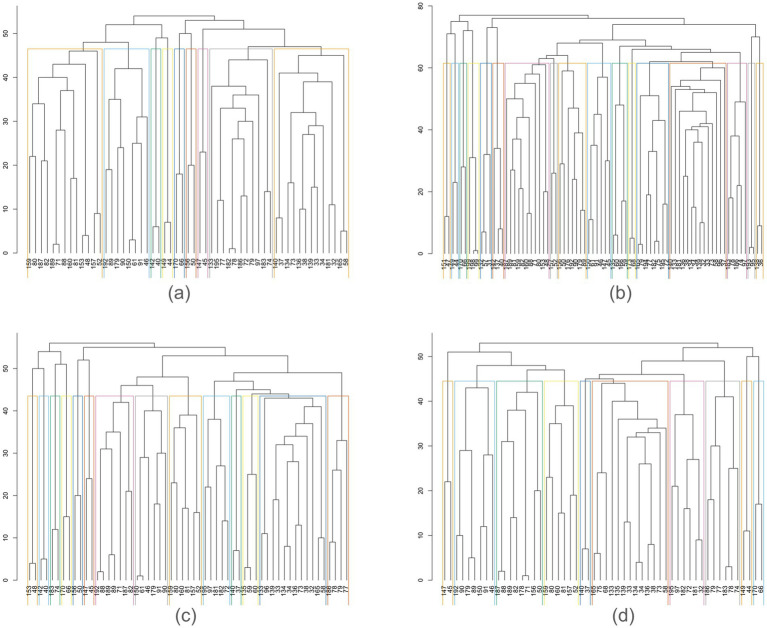
Hierarchical edge betweenness community. **(a)** Initiation, **(b)** Inhibition, **(c)** Shifting, **(d)** 2-back.

**Figure 8:**
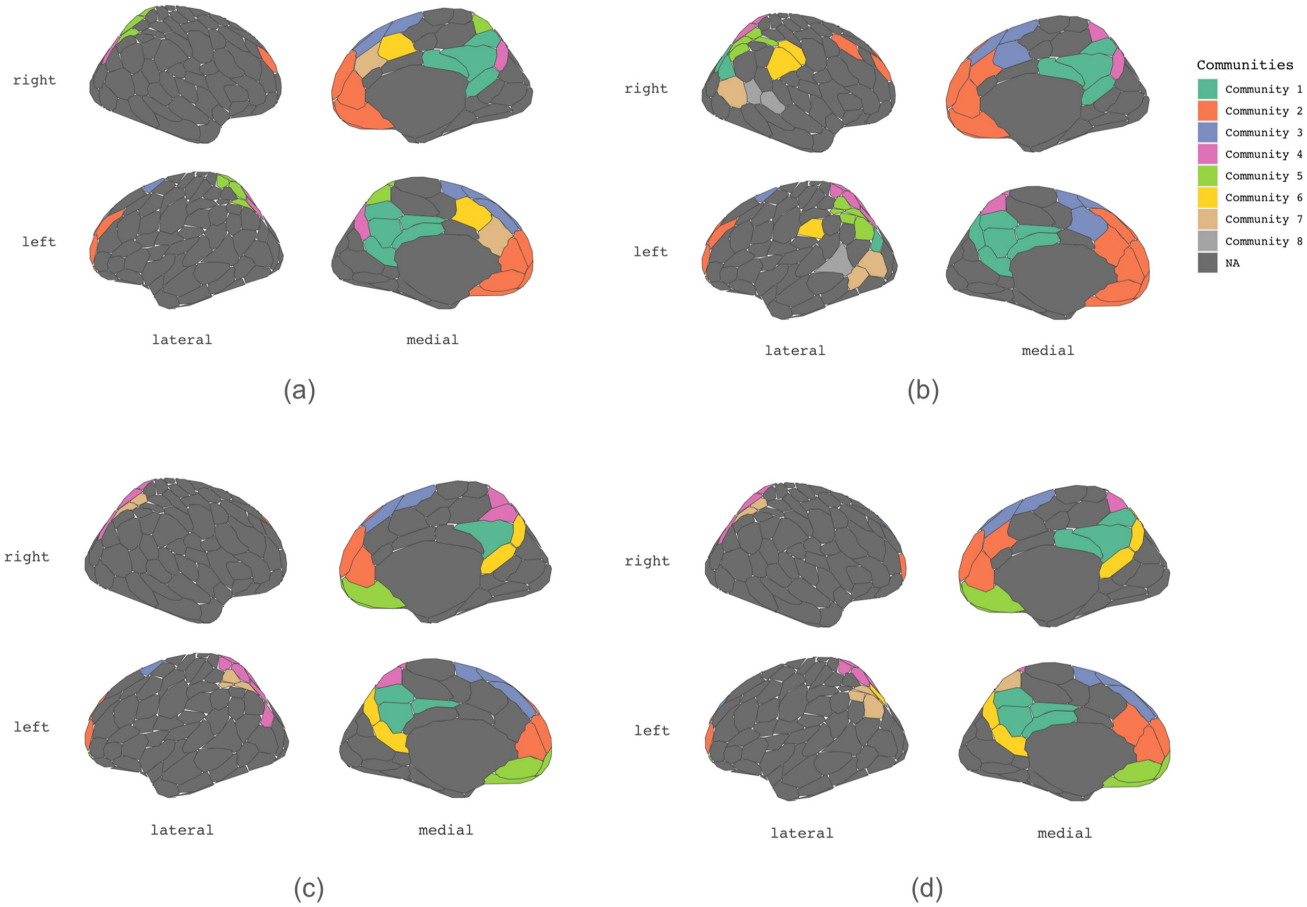
Main Louvain communities plotted on brain atlas. **(a)** Initiation, **(b)** Inhibition, **(c)** Shifting, **(d)** 2-back.

Tables 1–4 present each task’s node degree, strength, betweenness, and closeness. Table 6 and Figure 5 identify hubs with high strength and betweenness; Table 7 and Figure 6 highlight influencer nodes with high expected force. Figure 7 depicts the dendrogram for each graph’s edge betweenness community, and Tables 8–11 present the Louvain community for each graph. Figures 7, 8 illustrate the Louvain communities in the Schaefer atlas. Both algorithms yield comparable results.

**Table 6:** Hubs.

All 4 graphs	ROI	Schaefer node label	Cortical areas
	72	LH_ContC_pCun_1	LH Control Network Precuneus 1
	182	RH_ContC_pCun_2	RH Control Network Precuneus 2
	189	RH_DefaultA_PFCm_3	RH Default Network medial prefrontal 3

Initiation	ROI	Schaefer node label	Cortical areas
	33	LH_DorsAttnA_SPL_2	LH Dorsal Attention Network Superior Parietal Lobule 2
	72	LH_ContC_pCun_1	LH Control Network Precuneus 1
	73	LH_ContC_pCun_2	LH Control Network Precuneus 1
	159	RH_LimbicB_OFC_3	RH Limbic Network orbital frontal cortex 3
	182	RH_ContC_pCun_2	RH Control Network Precuneus 1
	186	RH_DefaultA_pCunPCC_1	LH Default Mode Network posterior cingulate cortex/precuneus 3
	189	RH_DefaultA_PFCm_3	RH Default Mode Network medial prefrontal cortex 3

Inhibition	ROI	Schaefer node label	Cortical areas
	72	LH_ContC_pCun_1	LH Control Network Precuneus 1
	77	LH_DefaultA_pCunPCC_1	LH Default Mode Network posterior cingulate cortex/precuneus 1
	88	LH_DefaultB_PFCd_1	LH Default Mode Network dorsal prefrontal cortex 1
	90	LH_DefaultB_PFCd_3	LH Default Mode Network dorsal prefrontal cortex 3
	159	RH_LimbicB_OFC_3	RH Limbic Network orbital frontal cortex 3
	182	RH_ContC_pCun_2	RH Control Network Precuneus 1
	186	RH_DefaultA_pCunPCC_1	LH Default Mode Network posterior cingulate cortex/precuneus 3
	189	RH_DefaultA_PFCm_3	Right Default Mode Network medial prefrontal cortex 3

Shifting	ROI	Schaefer node label	Cortical areas
	72	LH_ContC_pCun_1	LH Control Network precuneus 1
	73	LH_ContC_pCun_2	LH Control Network precuneus 2
	79	LH_DefaultA_pCunPCC_3	LH Default Mode Network posterior cingulate cortex/precuneus 3
	134	RH_DorsAttnA_SPL_2	RH Dorsal Attention Network Superior Parietal Lobule 2
	136	RH_DorsAttnA_SPL_4	RH Dorsal Attention Network Superior Parietal Lobule 4
	182	RH_ContC_pCun_2	RH Control Network Precuneus 2
	189	RH_DefaultA_PFCm_3	RH Default Mode Network medial prefrontal cortex 3

Working memory	ROI	Schaefer node label	Cortical areas
	33	LH_DorsAttnA_SPL_2	LH Dorsal Attention Network Superior Parietal Lobule 2
	72	LH_ContC_pCun_1	LH Control Network precuneus 1
	73	LH_ContC_pCun_2	LH Control Network precuneus 2
	159	RH_LimbicB_OFC_3	RH Limbic Network orbital frontal cortex 3
	182	RH_ContC_pCun_2	RH Control Network Precuneus 2
	186	RH_DefaultA_pCunPCC_1	LH Default Mode Network posterior cingulate cortex/precuneus 3
	189	RH_DefaultA_PFCm_3	RH Default Mode Network medial prefrontal cortex 3

**Table 7:** Influencers.

Initiation	ROI	Schaefer node label	Cortical areas
	33	LH_DorsAttnA_SPL_2	LH Dorsal Attention Network Superior Parietal Lobule 2
	72	LH_ContC_pCun_1	LH Control Network precuneus 1
	73	LH_ContC_pCun_2	LH Control Network precuneus 2
	134	RH_DorsAttnA_SPL_2	RH Dorsal Attention Network Superior Parietal Lobule 2
	136	RH_DorsAttnA_SPL_4	RH Dorsal Attention Network Superior Parietal Lobule 4
	139	RH_DorsAttnB_PostC_3	RH Dorsal Attention Network Post Central gyrus (medial segment)
	159	RH_LimbicB_OFC_3	RH Limbic Network orbital frontal cortex 3
	181	RH_ContC_pCun_1	RH Control Network precuneus 1
	182	RH_ContC_pCun_2	RH Control Network precuneus 2
	186	RH_DefaultA_pCunPCC_1	LH Default Mode Network posterior cingulate cortex/precuneus 3
	189	RH_DefaultA_PFCm_3	RH Default Mode Network medial prefrontal cortex 3

Inhibition	ROI	Schaefer node label	Cortical areas
	32	LH_DorsAttnA_SPL_1	LH Dorsal Attention Network Superior Parietal Lobule 1
	33	LH_DorsAttnA_SPL_2	LH Dorsal Attention Network Superior Parietal Lobule 2
	58	LH_ContA_IPS_1	LH Control Network Inferial Parietal Sulcus 1
	72	LH_ContC_pCun_1	LH Control Network precuneus 1
	73	LH_ContC_pCun_2	LH Control Network precuneus 2
	96	LH_DefaultC_IPL_1	LH Default Network Inferior Parietal Lobule 1
	133	RH_DorsAttnA_SPL_1	RH Dorsal Attention Network Superior Parietal Lobule 1
	134	RH_DorsAttnA_SPL_2	RH Dorsal Attention Network Superior Parietal Lobule 2
	136	RH_DorsAttnA_SPL_4	RH Dorsal Attention Network Superior Parietal Lobule 4
	139	RH_DorsAttnB_PostC_3	RH Dorsal Attention Network Post Central gyrus (medial segment)
	159	RH_LimbicB_OFC_3	RH Limbic Network orbital frontal cortex 3
	181	RH_ContC_pCun_1	RH Control Network precuneus 1
	182	RH_ContC_pCun_2	RH Control Network precuneus 2
	186	RH_DefaultA_pCunPCC_1	LH Default Mode Network posterior cingulate cortex/precuneus 3
	189	RH_DefaultA_PFCm_3	RH Default Mode Network medial prefrontal cortex 3
	194	RH_DefaultC_IPL_1	RH Default Network Inferior Parietal Lobule 1

Shifting	ROI	Schaefer node label	Cortical areas
	32	LH_DorsAttnA_SPL_1	LH Dorsal Attention Network Superior Parietal Lobule 1
	33	LH_DorsAttnA_SPL_2	LH Dorsal Attention Network Superior Parietal Lobule 2
	34	LH_DorsAttnA_SPL_3	LH Dorsal Attention Network Superior Parietal Lobule 3
	58	LH_ContA_IPS_1	LH Control Network Inferial Parietal Sulcus 1
	72	LH_ContC_pCun_1	LH Control Network precuneus 1
	73	LH_ContC_pCun_2	LH Control Network precuneus 2
	133	RH_DorsAttnA_SPL_1	RH Dorsal Attention Network Superior Parietal Lobule 1
	134	RH_DorsAttnA_SPL_2	RH Dorsal Attention Network Superior Parietal Lobule 2
	135	RH_DorsAttnA_SPL_3	RH Dorsal Attention Network Superior Parietal Lobule 3
	136	RH_DorsAttnA_SPL_4	RH Dorsal Attention Network Superior Parietal Lobule 4
	139	RH_DorsAttnB_PostC_3	RH Dorsal Attention Network Post Central gyrus (medial segment)
	189	RH_DefaultA_PFCm_3	RH Default Mode Network medial prefrontal cortex 3

Working memory	ROI	Schaefer node label	Cortical areas
	33	LH_DorsAttnA_SPL_2	LH Dorsal Attention Network Superior Parietal Lobule 2
	38	LH_DorsAttnB_PostC_4	LH Dorsal Attention Network Post Central Gyrus 4
	58	LH_ContA_IPS_1	LH Control Network Inferial Parietal Sulcus 1
	72	LH_ContC_pCun_1	LH Control Network precuneus 1
	73	LH_ContC_pCun_2	LH Control Network precuneus 2
	134	RH_DorsAttnA_SPL_2	RH Dorsal Attention Network Superior Parietal Lobule 2
	136	RH_DorsAttnA_SPL_4	RH Dorsal Attention Network Superior Parietal Lobule 4
	139	RH_DorsAttnB_PostC_3	RH Dorsal Attention Network Post Central gyrus (medial segment)
	165	RH_ContA_IPS_1	RH Control Network Inferial Parietal Sulcus 1
	182	RH_ContC_pCun_2	RH Control Network Precuneus 2
	189	RH_DefaultA_PFCm_3	RH Default Mode Network medial prefrontal cortex 3

Common influencers	ROI	Schaefer node label	Cortical areas
	33	LH_DorsAttnA_SPL_2	LH Dorsal Attention Network Superior Parietal Lobule 2
	72	LH_ContC_pCun_1	LH Control Network precuneus 1
	73	LH_ContC_pCun_2	LH Control Network precuneus 2
	134	RH_DorsAttnA_SPL_2	RH Dorsal Attention Network Superior Parietal Lobule 2
	136	RH_DorsAttnA_SPL_4	RH Dorsal Attention Network Superior Parietal Lobule 4
	139	RH_DorsAttnB_PostC_3	RH Dorsal Attention Network Post Central gyrus (medial segment)
	189	RH_DefaultA_PFCm_3	RH Default Mode Network medial prefrontal cortex 3

**Table 8:** Louvain community and edge betweenness community for initiation graph.

Louvain community	Nodes	Edge betweenness community	Nodes
1	77, 79, 97, 78, 74, 186, 182, 195, 183	1	77, 79, 97, 72, 78, 74, 186, 182, 195, 183, 133
2	88, 80, 82, 52, 81, 48, 71, 189, 159, 187, 160, 157, 153	2	88, 80, 82, 52, 81, 48, 71, 189, 159, 187, 160, 157, 153
3	46, 90, 89, 91, 61, 150, 179, 192	3	46, 90, 89, 91, 61, 150, 179, 192
4	33, 72, 32, 134, 181, 133	4	33, 73, 38, 34, 32, 37, 58, 134, 139, 181, 140, 136, 165
5	73, 38, 34, 37, 58, 139, 140, 136, 165	5	45, 147
6	45, 147	6	50, 156
7	50, 156	7	66, 170
8	66, 170	8	44, 149
9	44, 149	9	40, 142
10	40, 142		

**Table 9:** Louvain community and edge betweenness community for inhibition graph.

Louvain community	Nodes	Edge betweenness community	Nodes
1	77, 79, 97, 72, 78, 74, 96, 186, 182, 195, 183, 133, 194	1	77, 79, 72, 75, 182, 195, 165, 194
2	88, 90, 80, 52, 82, 50, 81, 89, 48, 71, 189, 159, 187, 156, 160, 192, 157, 153, 176	2	88, 80, 82, 81, 48, 71, 189, 159, 187, 160, 153
3	46, 45, 91, 61, 179, 150, 147	3	33, 73, 38, 34, 32, 58, 96, 37, 134, 139, 181, 136, 140, 133
4	33, 73, 38, 34, 32, 37, 134, 139, 181, 136, 140	4	90, 50, 89, 179, 156, 192, 176
5	58, 59, 75, 60, 68, 165, 135, 173, 166	5	46, 45, 91, 61, 150, 147
6	66, 170	6	97, 78, 74, 186, 183
7	40, 142, 137	7	52, 157
8	44, 149	8	66, 170
9	31, 57, 132	9	40, 142, 137
10	99, 199, 198	10	44, 149
11	47, 151	11	59, 60, 135, 166
12	36, 138	12	31, 57, 132
13	95, 193	13	99, 199, 198
		14	47, 151
		15	36, 138
		16	68, 173
		17	95, 193

**Table 10:** Louvain community and edge betweenness community for shifting graph.

Louvain community	Nodes	Edge betweenness community	Nodes
1	77, 79, 78, 186	1	77, 79, 78, 186
2	88, 82, 89, 71, 189, 187, 192	2	88, 82, 89, 71, 189, 187, 192
3	90, 46, 91, 61, 179, 150	3	73, 33, 34, 38, 58, 32, 96, 139, 134, 136, 133, 165
4	73, 33, 34, 38, 32, 96, 37, 139, 134, 182, 136, 140, 133	4	90, 46, 91, 61, 179, 150
5	80, 52, 81, 159, 160, 157	5	45, 147
6	72, 97, 181, 195	6	72, 97, 182, 181, 195
7	58, 60, 59, 165, 135	7	80, 52, 81, 159, 160, 157
8	50, 156	8	50, 156
9	45, 147	9	66, 170
10	66, 170	10	74, 183
11	74, 183	11	60, 59, 135
12	40, 142	12	37, 140
13	48, 153	13	40, 142
		14	48, 153

**Table 11:** Louvain community and edge betweenness community for 2-back graph.

Louvain community	Nodes	Edge betweenness community	Nodes
1	77, 79, 78, 74, 186, 182, 183	1	88, 50, 82, 71, 189, 156, 187, 178
2	88, 50, 82, 71, 189, 156, 187, 178	2	77, 79, 78, 74, 186, 183
3	90, 46, 89, 91, 179, 150, 192	3	73, 33, 38, 34, 58, 68, 75, 139, 134, 136, 165, 135, 133
4	33, 38, 34, 37, 139, 136, 140, 133	4	90, 46, 89, 91, 179, 150, 192
5	80, 52, 81, 159, 157, 160	5	72, 97, 32, 182, 181, 195
6	72, 97, 32, 134, 181, 195	6	80, 52, 81, 159, 157, 160
7	73, 58, 68, 75, 165, 135	7	45, 147
8	45, 147	8	66, 170
9	66, 170	9	44, 149
10	44, 149	10	37, 140

A comparative analysis of graph network measures across subdomains of executive functioning, including initiation, cognitive inhibition, mental shifting, and working memory, unveiled nuanced variations and commonalities in the organization and functional connectivity patterns. Specific brain regions consistently emerged as prominent hubs (Table 6), facilitating information exchange within the graph of all four subdomains of executive function. These regions include the bilateral precuneus (LH_ContC_pCun_1, RH_ContC_pCun_2) and the right medial prefrontal cortex (DMN) (RH_DefaultA_PFCm_3). Notably, these regions exhibited high node strength and betweenness centrality and are connected to each other, indicating their pivotal role in facilitating executive functioning processes. Closeness centrality analysis revealed that several nodes in each graph exhibit a closeness of 1 (Table 4), yet they form isolated subnetworks. Nodes with the following highest closeness values are notably low, suggesting their relative distance from other nodes regarding functional connectivity. This implies reduced efficiency in transmitting information or influence across the broader brain network.

A Kolmogorov–Smirnov test compared the observed network properties with those of randomly generated networks, revealing significant differences in degree distributions (Table 12). Considerable variations were identified among the shared ROIs across the four subdomains. Utilizing a Kruskal-Wallis test, we observed a chi-squared value of 21.634 and a *p*-value of 7.772e-05, indicating statistically significant discrepancies in strength across the four tasks. As depicted using the Wilcoxon rank sum test (Table 13), notable disparities in the strength of common nodes (ROIs) were evident, particularly between inhibition and the other functions. Conversely, no statistically significant distinctions between initiation, shifting, and the 2-back tasks were observed (Table 14).

**Table 12:** Degree distribution.

Initiation		Inhibition		Shifting		2-back	
Degree	Distribution	Degree	Distribution	Degree	Distribution	Degree	Distribution
1	0.4	1	0.3461538	1	0.4736842	1	0.3518519
2	0.2363636	2	0.2179487	2	0.1929825	2	0.2962963
3	0.1818182	3	0.1153846	3	0.1403509	3	0.1481481
6	0.0545455	4	0.0897436	4	0.1403509	4	0.1296296
5	0.0545455	5	0.1153846	5	0.0175439	5	0.037037
7	0.0181818	6	0.0512821	6	0.0350877	6	0.0185185
		7	0.0128205			7	0.0185185
		8	0.0512821				

**Table 13:** Pairwise comparisons using the Wilcoxon rank sum test.

Tasks	Initiation	Inhibition	Shifting
Inhibition	0.0026	–	–
Shifting	1	0.0154	–
2back	1	0.0001	0.9116

**Table 14:** Kolmogorov–Smirnov test results.

Initiation		Inhibition		Shifting		2-back	
D	*p*-value	D	*p*-value	D	*p*-value	D	*p*-value
0.85455	2.01E-05	0.96154	3.11E-09	0.87719	2.65E-05	0.87037	8.14E-06

Unique regions of interest (ROIs) specific to each task (Table 15) are also analyzed. ROIs 194, 135, 96, and 75 exhibit high degrees but low strength. The inhibition graph possesses the highest number of distinct ROIs compared to the other graphs and appears to be the most distinctive among the four. Kruskal-Wallis rank sum tests were performed for a list of hubs from each graph, resulting in a *p*-value of 0.7965. This indicates no significant difference in the hubs among the graphs.

**Table 15:** Distinct nodes across tasks.

Node	Initiation	Inhibition	Shifting	Two back
31	NA	1	NA	NA
36	NA	1	NA	NA
40	1	2	1	NA
44	1	1	NA	1
47	NA	1	NA	NA
48	2	2	1	NA
57	NA	1	NA	NA
59	NA	2	1	NA
60	NA	1	1	NA
61	1	1	1	NA
68	NA	1	NA	1
75	NA	4	NA	1
95	NA	1	NA	NA
96	NA	5	1	NA
99	NA	2	NA	NA
132	NA	2	NA	NA
135	NA	3	4	2
137	NA	1	NA	NA
138	NA	1	NA	NA
142	1	1	1	NA
149	1	1	NA	1
151	NA	1	NA	NA
153	1	1	1	NA
166	NA	1	NA	NA
167	NA	NA	2	NA
169	NA	NA	1	NA
173	NA	2	NA	NA
175	NA	NA	1	NA
176	NA	1	NA	NA
178	NA	NA	NA	1
193	NA	1	NA	NA
194	NA	5	NA	NA
198	NA	1	NA	NA
199	NA	1	NA	NA

Unique to the cognitive inhibition graph are regions LH_DefaultA_pCunPCC_1 (left posterior cingulate cortex/precuneus), LH_DefaultB_PFCd_1 (left dorsal prefrontal cortex 1), and LH_DefaultB_PFCd_3 (left dorsal prefrontal cortex 3) (ROIs 77, 88, and 90), which emerge as hubs. Conversely, in the mental shifting graph, regions LH_DefaultA_pCunPCC_3 (left posterior cingulate cortex/precuneus 3), RH_DorsAttnA_SPL_2 (right superior parietal lobule 2), and RH_DorsAttnA_SPL_4 (right superior parietal lobule 4)(ROIs 134, 136, and 79) assume hub roles. Regions unique to cognitive inhibition and mental shifting (Table 15) were detected. In the inhibition graph, ROIs 75, 96, and 194 (LH_DefaultA_IPL_1, LH_DefaultC_IPL_1, and RH_DefaultC_IPL_1), specifically, bilateral inferior parietal lobule, were identified as key regions of connectivity. In contrast, the shifting graph exhibited uniquely high connections involving ROI 135 (RH_DorsAttnA_SPL_3), the right superior parietal lobule.

Furthermore, each executive subdomain was associated with distinct influencers (Table 12). Notably, many of the identified influencers also function as hubs. Additionally, initiation function is influenced by ROIs 136, 134, and 139 (RH Dorsal Attention Network Superior Parietal Lobule 2, RH Dorsal Attention Network Superior Parietal Lobule 4, and RH Dorsal Attention Network Post Central gyrus (medial segment)) as influencers. Cognitive inhibition is influenced by several regions of interest (ROIs) in the human brain, including:

1. Left Hemisphere: Superior Parietal Lobule 1 (LH Dorsal Attention Network), Inferior Parietal Sulcus 1 (LH Control Network), and Inferior Parietal Lobule 1 (LH Default Network).
2. Right Hemisphere: Superior Parietal Lobule 1 (RH Dorsal Attention Network), Superior Parietal Lobule 4 (RH Dorsal Attention Network), Post Central Gyrus (medial segment) (RH Dorsal Attention Network), Precuneus 1 (RH Control Network), and Inferior Parietal Lobule 1 (RH Default Network).
3. Mental shifting requires influences from ROIs 32, 33, 34, 58, 133, and 135 (LH Dorsal Attention Network Superior Parietal Lobule 1, LH Dorsal Attention Network Superior Parietal Lobule 2, LH Dorsal Attention Network Superior Parietal Lobule 3, LH Control Network Inferior Parietal Sulcus 1, RH Dorsal Attention Network Superior Parietal Lobule 1, and RH Dorsal Attention Network Superior Parietal Lobule 3).
4. Working memory requires influences from ROIs 38, 58, and 165 (LH Dorsal Attention Network Post Central Gyrus 4, LH Control Network Inferior Parietal Sulcus 1, and RH Control Network Inferior Parietal Sulcus 1).

Brain graphs with a short characteristic path length are believed to integrate information more efficiently between nodes (Paldino et al., 2016). In contrast, inhibition graphs exhibit a relatively long characteristic path length of 5.1681 compared to the initiation, mental shifting, and working memory graphs (3.348269, 3.9143, and 3.9273, respectively). Initiation has a lower assertiveness value, indicating a more neutral or distributed balance, balancing local and global connectivity. However, it might rely more on specific hubs for overall functionality, making it vulnerable to hub damage, as seen in conditions like stroke or traumatic brain injury. Global efficiency is inversely proportional to the topological distance between nodes and is typically interpreted as a measure of the capacity for parallel information transfer and integrated processing (Bullmore and Sporns, 2009). The observation that all graphs exhibit low to moderate levels of global efficiency suggests that the brain regions are not highly interconnected, thereby limiting the efficiency of information transmission across the network. Furthermore, the degree distribution (Table 5) exhibited characteristics of an exponentially truncated power law distribution. In other words, most nodes have relatively low degrees, while some have extremely high degrees.

The clustering coefficient provides insight into the local connectivity of nodes within a network, reflecting the extent to which neighboring nodes are interconnected (Bullmore and Sporns, 2009). It assesses the prevalence of clustered connections among nearby nodes, indicating the likelihood of forming local clusters or communities. Path transitivity evaluates the number of local detours along a path, contributing to understanding how efficiently information flows within the network. Graphs with a high small-world value exhibit densely clustered local connections and optimal long-range connections, facilitating efficient information processing at minimal cost (Bassett and Bullmore, 2006; Bullmore and Sporns, 2009). The clustering coefficient, transitivity, and small-worldedness sigma of 0 indicate a decentralized structure. However, modularity values of all four graphs suggest the presence of distinct communities across all subdomains of executive function, highlighting the absence of local clustering. Indeed, the community detection analysis unveils a rich modular structure within each graph (Figures 3, 4; Tables 8–11).

## Discussion and clinical implications

The evolution of graph theory in cognitive neuroscience has provided valuable insights into the intricate connections within the human brain, offering a robust framework for understanding cognitive processes and their neural underpinnings (Bullmore and Sporns, 2009; Farahani et al., 2019; Medaglia, 2017; Medaglia et al., 2015). Executive functioning, essential for daily activities (Zelazo et al., 2004), encompasses various cognitive processes. This study enhances our understanding of executive functioning in healthy adults by identifying key hub/influencer regions and analyzing local and global properties of subdomains of executive functioning, namely, initiation, cognitive inhibition, mental shifting, and working memory.

### Hubs and influencers

Our hypothesis that specific brain regions will serve as critical hubs or “influencers” across these tasks was confirmed. The precuneus and right medial prefrontal cortex (mPFC) emerged as crucial hubs for all four subdomains of executive function. Our findings also support previous research highlighting the dorsolateral prefrontal cortex (DLPFC), anterior cingulate cortex (ACC), and parietal regions as key components of executive function.

Both the precuneus and mPFC have been identified as integral components of the default mode network (DMN), which is typically active during rest and internally directed thought (Cavanna and Trimble, 2006; Yeager et al., 2022; Friedman and Robbins, 2022; Jobson et al., 2021; Menon and D’Esposito, 2022). The DMN deactivates during cognitively demanding tasks, allowing for more focused information processing (Jin et al., 2012; Leech and Sharp, 2014; Salgado-Pineda et al., 2021; Billette et al., 2022; Xu et al., 2019).

In our study, the precuneus exhibited connectivity with the posterior cingulate cortex (PCC), which deactivates alongside the precuneus during executive function tasks (Raichle, 2015), as well as with the inferior and superior parietal cortices, which exhibit task-dependent activation levels (Yeo et al., 2015). Similarly, the right mPFC showed strong connectivity with other prefrontal regions, which are generally activated during executive functions.

Given their role as hubs and “influencers,” the mPFC and precuneus likely regulate network-wide activity, influencing when to engage or suppress cognitive processes depending on task demands. Dysfunction in these regions is associated with attention deficits, impaired self-referential thinking, and decision-making difficulties and has been linked to neurological disorders such as Alzheimer’s disease, schizophrenia, and depression (Buckner et al., 2009; Menon, 2011). These findings highlight the potential of these regions as targets for neuromodulation techniques, such as transcranial magnetic stimulation (TMS), to enhance executive function in individuals affected by stroke, neurodegeneration, or cognitive impairments (Guse et al., 2010).

While the literature on the right hemisphere is less extensive, surgical mapping studies have indicated the involvement of the right ventromedial prefrontal cortex (vmPFC) and orbital frontal areas in facial emotion recognition and theory of mind (Bernard et al., 2018). Our findings suggest a hub and influencer role for the right mPFC in executive functioning, contributing to our understanding of right hemisphere involvement in cognitive processes.

In addition to hub regions shared by all four subdomains of executive functions, our findings also identified unique hubs for cognitive inhibition as the left posterior cingulate cortex/precuneus and the left dorsal prefrontal cortex. Moreover, the mental shifting function relied on hub regions such as the left posterior cingulate cortex/precuneus and the right superior parietal lobule. The superior parietal lobule had previously been studied for the function of attentional shifting (Wang et al., 2014). Interestingly, bilateral inferior parietal lobules exhibited high connectivity in cognitive inhibition, aligning with the previous study on the parietal cortex’s contribution to inhibitory processes (Kolodny et al., 2017). Potential treatment strategies could be developed by targeting these regions for executive function disorders such as ADHD and inhibitory control disorders.

In addition to the “influencer” regions shared by all four subdomains, the bilateral superior parietal lobule and the right post-central gyrus (medial segment) are also identified as “influencers.” The superior parietal lobule is considered to play a pivotal role in numerous cognitive functions (Wang et al., 2014). The bilateral inferior parietal sulcus (IPS) plays an “influencer” role in cognitive inhibition, mental shifting, and working memory. This is similar to another study suggesting that IPS plays an essential role in executive functioning, particularly inhibition (Osada et al., 2019). Working memory appears to have a segment of the left post-central gyrus (DAN) as an “influencer,” similar to the findings on working memory among early Parkinson’s patients (Alsakaji et al., 2021). A segment of the right inferior parietal lobule (IPSL) appears to be an “influencer” of cognitive inhibition, which could be attributed to its involvement in visual attention (Corbetta and Shulman, 2002). As mentioned, “influencers” are more resilient to network reorganization. Therefore, these regions could be potential targets for the treatment of executive function deficits post-TBI, seizure disorders, or post-tumor resection.

### Efficiency and communities

Unlike our hypothesis, each subdomain of the executive function showed similar network features except for inhibition. We also did not find increased connectivity in control-related regions or higher modularity for working memory. However, the cognitive inhibition graph exhibits slightly longer characteristic path lengths and greater overall region involvement than its counterparts, such as the parieto-occipital cortex (DAN) and temporal–parietal regions. These findings suggest cognitive inhibition involves a brain network organization that prioritizes specialized information transfer between regions, emphasizing the distinct nature of inhibitory control processes within the executive network. While this may result in less efficient overall network function, it may also reflect a more targeted and specialized approach to cognitive processing in inhibition.

Conversely, initiation, mental shifting, and working memory have shorter path lengths, which could minimize the metabolic cost associated with routing action potentials across axons and synaptic contacts and, hence, could provide faster, more direct, and less noisy information transfer (Bullmore and Sporns, 2009). Our analysis of graph metrics collectively implies a decentralized and modular functioning organization, wherein information processing occurs across distributed networks rather than being confined to specific localized regions.

Distinct subsystems of communities consistently emerge across four subdomains, prominently featuring medial parietal regions and the posterior medial frontal area across all four executive functioning subdomains. Echoing established findings, the medial prefrontal region exhibits a recurring presence during executive tasks yet notably delineates into two discernible subsystems: the dorsomedial prefrontal cortex (dmPFC) and ventromedial prefrontal cortex (vmPFC), particularly during mental shifting and working memory processes. This partition may stem from the dmPFC’s primary connections to the neocortex, while the vmPFC primarily interfaces with the limbic system (Jobson et al., 2021).

### Vulnerability and resilience

Unlike our hypothesis, we did not find significant differences in their topological properties across tasks related to these four subdomains of executive functions. Our findings support a more distributed network topology for all four subdomains of executive functions. A distributed network, which does not rely heavily on single central components, offers resilience to random damage, as observed in the human brain’s robust response to lesions (Achard et al., 2006; Aerts et al., 2016), especially in a pediatric population (Guan et al., 2024). This resilience provides a framework for understanding the brain’s ability to maintain cognitive functions even after injury.

On the other hand, The vulnerability of hub regions to targeted damage highlights their critical role. Lesions in these hubs, such as those occurring in stroke or traumatic brain injury, can significantly impair executive functioning. Initiation stood out as it exhibits a low assortativity value, indicating a distributed network with balanced local and global connectivity but relying on hubs and communities (mPFC). Therefore, it could be more vulnerable than the other executive functions. Indeed, motivational and initiation deficits frequently occur in individuals with acquired brain injury, where prefrontal areas are more vulnerable (Palmisano et al., 2020). This understanding could guide clinical interventions, such as targeted behavioral therapy or deep brain stimulation, to restore function in affected regions (Aerts et al., 2016). Moreover, alterations in global network topology observed in conditions like Alzheimer’s disease, multiple sclerosis, and epilepsy suggest that these pathologies may function as “disconnection syndromes,” where disrupted connectivity underlies cognitive deficits (Guye et al., 2010). Understanding brain networks’ distributed and resilient nature can inform rehabilitation strategies to leverage intact pathways to compensate for lost functions. Further research is needed to explore how these network characteristics evolve across different conditions and stages of brain damage.

Recent studies have further highlighted the clinical implications of distributed network topology in executive functions. For instance, research shows that the topological properties of the frontoparietal network (FPN) and default mode network (DMN) are associated with executive function performance across the lifespan, with the DMN showing greater sensitivity to age-related changes (Menardi et al., 2024). Additionally, alterations in network topology have been observed in patients with mild cognitive impairment (MCI), suggesting that changes in network organization could serve as imaging markers for early diagnosis and intervention before Alzheimer’s disease onset (Xue et al., 2024).

Recent research has also emphasized the role of hub regions in neurological disorders. For example, in Parkinson’s disease, the spread of *α*-synuclein through connected brain regions leads to neuronal loss and network disruptions, with hub regions playing a significant role in this process (Frigerio et al., 2024). Understanding the involvement of hub regions is becoming increasingly important for clinical practice, as these hubs are critical for maintaining normal brain function and enabling complex behavior (Stam, 2024). These findings reinforce the importance of network topology in developing targeted interventions and rehabilitation strategies for various neurological conditions.

## Limitations and future direction

Several limitations exist besides the small sample size and exclusive focus on the brain’s cortical areas. A key concern is that the cognitive paradigms used may not adequately capture the complex nuances of the four subdomains of executive functioning: initiation, inhibition, mental shifting, and working memory. While these paradigms provide valuable insights, they may not fully reflect the intricacies of these cognitive processes. This limitation underscores the need for future research to employ various cognitive tasks for a more thorough assessment of executive functioning.

Additionally, while graph analysis yields important insights into the dynamics of brain networks associated with cognitive tasks, several significant limitations exist. Reducing the brain into nodes and edges oversimplifies its inherent complexity, and the decisions made regarding the parcellation schemes and network construction parameters can significantly impact the results. Factors such as the spatial and temporal resolution of neuroimaging data, individual variability, and subjective thresholding methods introduce potential confounding variables. Furthermore, the cross-sectional nature of the analysis limits our understanding of how these dynamics change over time. Interpreting graph metrics concerning neural processes also requires caution due to their context-dependent nature. Addressing these limitations is critical for enhancing our understanding of brain network organization and functionality.

Despite these constraints, the study significantly contributes to our understanding of how brain networks support various cognitive processes. Future research should explore the subdomains of executive functioning with diverse cognitive paradigms, expand data collection to include subcortical activities and examine the complex interplay between brain networks and cognitive processes.

## Conclusion

This study enhances our understanding of executive functioning by identifying key hubs, influencers, and communities while examining local and global network characteristics across four subdomains of executive function. Central areas such as the bilateral precuneus and the right medial prefrontal area are indispensable for integrating, transmitting information, and regulating activities within distributed networks, rendering them essential to executive functioning. Damage to these hubs can disrupt the executive function network.

Rehabilitation strategies can capitalize on neuroplasticity to preserve or enhance the functionality of these critical hubs. Techniques such as transcranial magnetic stimulation (TMS) or transcranial direct current stimulation (tDCS) may target these regions to facilitate the restoration of connectivity and enhance cognitive outcomes. Furthermore, task-specific cognitive training designed to activate these hubs can promote network reorganization, enabling compensatory pathways to develop and improve recovery.

The distributed nature of executive function networks also suggests resilience in cognitive recovery. Even when a hub is compromised, strengthening other regions or connections within the network may mitigate deficits. Incorporating insights into hub functionality facilitates more targeted and effective rehabilitation, improving outcomes for individuals with brain injuries.

Moreover, this study elucidates distinct hubs and influencers specific to each executive function subdomain, underscoring the unique characteristics of these cognitive processes. Consistent with prior research, the bilateral precuneus is reaffirmed as a pivotal hub and influencer in executive functioning. Our finding on the central role of the right mPFC in executive functioning could point to a new direction in research in the right hemisphere.

The resilience of distributed brain networks to damage holds significant implications for conditions such as stroke and traumatic brain injury, guiding interventions aimed at preserving executive function. Further research is necessary to elucidate how network organization adapts to various types of brain damage, including epilepsy and neurodegenerative diseases, and to develop targeted therapeutic strategies that enhance recovery.

## Data Availability

Publicly available datasets were analyzed in this study. This data can be found at: https://pubmed.ncbi.nlm.nih.gov/34877370/.

## Author’s note

R Studio was used as an integrated development environment for R programming. The data were processed and analyzed using various R packages, including the `sqldf` package for SQL-like data manipulation (Grothendieck, 2007), the `brainGraph` package for brain network analysis (Watson, 2015), the `igraph` package for graph theory analysis (Csárdi et al., 2006), the `ggraph` package for advanced graph visualization (Pedersen, 2021), the `ggplot2` package for creating plots (Pedersen and RStudio, 2024), and the `brainconn` package for connectivity analysis (Chopra, n.d.). The analysis reports were generated using the `knitr` package for dynamic report generation (Xie, 2025), and tables were formatted using the `kableExtra` package (Zhu, 2024).
