# Supplementary figures and images for: Hubs, influencers, and communities of executive functions: a task-based fMRI graph analysis
Source: Front Hum Neurosci. 2025 Aug 25;19:1525497. doi: 10.3389/fnhum.2025.1525497 (PMC12415012; doi:10.3389/fnhum.2025.1525497)

# Supplementary Material

### Figure 1. Chord Diagram of each graph

##
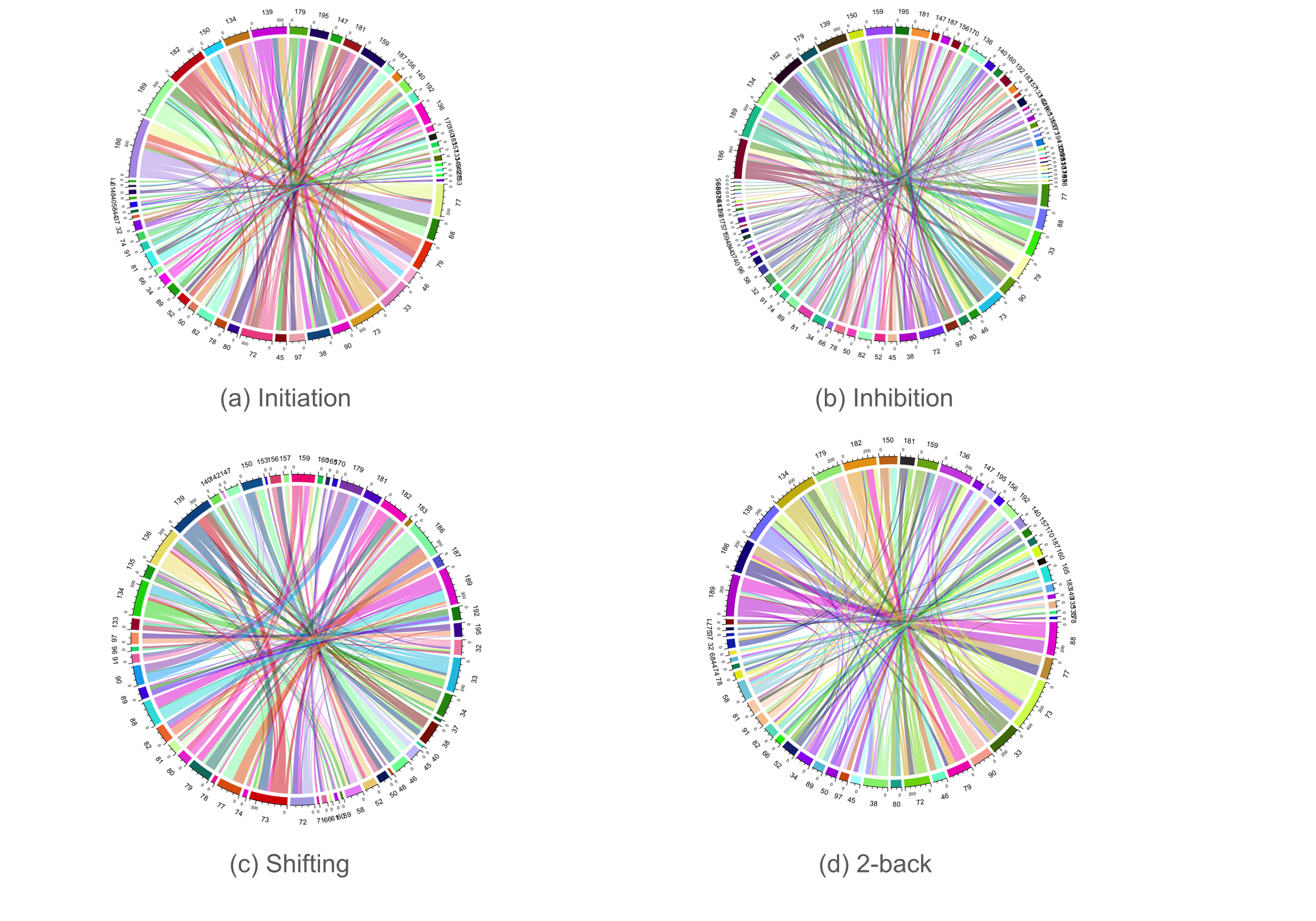

Supplement: Supplementary file 1 [file Data_Sheet_1.docx]
